# Simplified Large-Scale Refolding, Purification, and Characterization of Recombinant Human Granulocyte-Colony Stimulating Factor in *Escherichia coli*

**DOI:** 10.1371/journal.pone.0080109

**Published:** 2013-11-04

**Authors:** Chang Kyu Kim, Chi Ho Lee, Seung-Bae Lee, Jae-Wook Oh

**Affiliations:** 1 Department of Animal Biotechnology/Animal Resources Research Center, College of Animal Bioscience and Biotechnology, Konkuk University, Seoul, Korea; 2 Department of Food Science and Biotechnology of Animal Resources, College of Animal Bioscience and Biotechnology, Konkuk University, Seoul, Korea; 3 Division of Animal Resources and Life Science, Sangji University, Wonju, Korea; Tecnologico de Monterrey, Mexico

## Abstract

Granulocyte-colony stimulating factor (G-CSF) is a pleiotropic cytokine that stimulates the development of committed hematopoietic progenitor cells and enhances the functional activity of mature cells. Here, we report a simplified method for fed-batch culture as well as the purification of recombinant human (rh) G-CSF. The new system for rhG-CSF purification was performed using not only temperature shift strategy without isopropyl-l-thio-β-d-galactoside (IPTG) induction but also the purification method by a single step of prep-HPLC after the pH precipitation of the refolded samples. Through these processes, the final cell density and overall yield of homogenous rhG-CSF were obtained 42.8 g as dry cell weights, 1.75 g as purified active proteins, from 1 L culture broth, respectively. The purity of rhG-CSF was finally 99% since the isoforms of rhG-CSF could be separated through the prep-HPLC step. The result of biological activity indicated that purified rhG-CSF has a similar profile to the World Health Organization (WHO) 2^nd^ International Standard for G-CSF. Taken together, our results demonstrate that the simple purification through a single step of prep-HPLC may be valuable for the industrial-scale production of biologically active proteins.

## Introduction

Biosimilars are biologic medical products that are made by living organisms using recombinant DNA technology [1,2]. One of these biosimilars is granulocyte-colony stimulating factor (G-CSF). G-CSF is known to regulate the proliferation of neutrophil progenitor cells and their differentiation to granulocytes and functionally activates mature neutrophils [3,4]. It is produced in response to specific stimulation by a variety of cells including macrophages, fibroblasts, endothelial cells, and bone marrow stroma [5,6]. Currently, G-CSF is used clinically to facilitate hematopoietic recovery after bone marrow transplantation [7,8] or cancer chemotherapy [9].

The gene of G-CSF is existed on chromosome 17 that encodes two protein products due to alternative splicing: isoform A composed of 177 amino acids and isoform B of 174 amino acids. Isoform A contains an additional three residues (Val-Ser-Gln) inserted after Leu35 in isoform B. Isoform B has greater biological activity and stability than those of isoform A [1]. Thus, human (h) G-CSF, isoform B, is mainly targeted for cloning and expression. The G-CSF is an 18.8-kDa glycoprotein that has 5 cysteine residues as well as 2 intra-molecular disulfide bonds that are indispensable to its biological activity [10,11]. Recombinant (r) hG-CSF produced by *Escherichia coli* (*E. coli*) has similar biological activity to that of the native human protein, but differs in that it contains an N-terminal methionine residue and is not glycosylated [12]. The expression of rhG-CSF in *E. coli* frequently results in the formation of insoluble aggregates, which are called inclusion bodies (IBs). These aggregates are solubilized in a buffer containing chaotropic agents such as urea [13,14] or guanidine [15]. Thus, it is important to renature the denatured forms to be biologically active forms with apposite 3D structure [16,17]. In addition, to gain rhG-CSF of high quality, isoforms that are detected in reverse phase (RP)-high-performance liquid chromatography (HPLC) step during rhG-CSF purification are needed to separate through additional methods [18,19], such as ion exchange chromatography [20], hydrophobic interaction chromatography [21], and size exclusion chromatography (SEC) [22].

In this study, we describe the importance of the high cell density culture using a temperature shift strategy for rhG-CSF expression of high quality without isopropyl-l-thio-β-d-galactoside (IPTG) induction in *E. coli*. As well, simplified refolding and purification processes to separate rhG-CSF isoforms for pilot scale production are introduced.

## Materials and Methods

### Bacterial strain and plasmid

*E. coli* JM109 (*endA1 recA1 gyrA96 hsdR17 relA1 supE44* *thiΔ* (*lac-proAB*) *F'* [*traD*36 *proAB* ^*+*^ *lacI* ^*q*^ *lacZ ΔM*15]) was purchased from Stratagene (La Jolla, CA). The expression plasmid pPT, in which the *g-csf* (*isoform B*) gene was inserted into the *Nde*I and *Hind*III sites of the vector, was used for protein production as previously described [13,23].

### Fed-batch culture

Luria-Bertani (LB) containing 50 µg/mL ampicillin was used for the seed culture. The production medium for high cell density culture was composed of 7.5 g/L glucose, 5 g/L yeast extract, 7.5 g/L KH_2_PO_4_.12H_2_O, 3 g/L MgSO_4_.7H_2_O, trace elements, 100 µg/mL ampicillin, and 0.2 g/L antifoaming agent. Feeding medium A (C-source) contained 400 g/L glucose, 20 g/L MgSO_4_.7H_2_O, 20× trace elements, and 1 g/L ampicillin. Feeding medium B (N-source) contained 400 g/L yeast extract, 1 g/L ampicillin, and 0.5 g/L antifoaming agent. Seed culture was carried out at 37°C, 250 rpm, for 8–10 h. Main culture was performed in a 300-L fermenter (D 300; B. Braun, Melsungen, Germany) with a starting volume of 120 L. The culture was started under the following conditions: 30°C, >30% saturation dissolved oxygen (DO), 1 vvm air flow, internal pressure 0.5 kgf/cm^2^, and 200 rpm. The culture broth was maintained at pH 7.0 using ammonia water. According to the decreased value of DO, the agitation speed, air flow rate, and internal pressure were increased up to 500 rpm, 2 vvm, 1.0 kgf/cm^2^ respectively. The addition of feeding media A and B was controlled by a peristaltic pump to maintain a 0.05% glucose concentration in the culture broth. The initial culture temperature was at 30°C and increased in a stepwise manner to 37°C as previously described [24].

### IBs solubilization and refolding of rhG-CSF

The cultivated cells were harvested using tubular type continuous centrifuge (ASM260, TOMOE Engineering, Tokyo, Japan) at 10,000 × *g* for 20 min. The cell pellets were suspended in a lysis buffer (0.1 M Tris-HCl, 20 mM EDTA, pH 7.9) and disrupted by a homogenizer (Picomax MN 300-25P, Micronox, Seongnam, Korea) at 20,000 psi. The IBs were washed twice with buffer A (20 mM. Tris-HCl, 5 mM EDTA, 0.5% Triton X-100, pH 7.9), solubilized in buffer B [8 M urea and 50 mM glycine, 80 µM β-mercaptoethanol (pH 8.0)], and refolded in buffer C [0.8 M urea and 50 mM glycine, 8 µM β-mercaptoethanol (pH 8.0)]. The refolding was carried out at 25°C for 15 h. After refolding, the solution was adjusted to pH 5.5 with phosphoric acid to separate contaminating *E. coli* proteins followed by centrifugation at 10,000 × *g* for 20 min, and then the supernatant was concentrated 20-fold by ultrafiltration system (Sartocon, Sartorius, Goettingen, Germany) with MWCO 10K cassettes.

### rhG-CSF purification

#### Semi(s)-preparative (prep) high-performance liquid chromatography (HPLC)

The concentrated supernatant (10 mg) was loaded on a sp-HPLC column (Vydac Protein C_4_ # 214 TP 1010, 250 mm × 10 mm), and collected the fractions containing rhG-CSF. Each fraction was diluted in distilled water (pH 3.5) and concentrated by the Amicon ultrafiltration system. Elution was carried out at a flow rate of 2 mL/min at 40°C with a linear gradient of acetonitrile (ACN, 47.25–76.5%) for 140 min using solvent A (22.5% ACN and 0.1% TFA) and solvent B (90% ACN and 0.1% TFA). The eluted sample was detected at 280 nm absorbance.

#### Prep-HPLC

All experimental conditions for sample preparation and performance of prep-HPLC were similar to that of semi-prep-HPLC. The Prep-HPLC System (R&S Technology, RI, USA) was performed using Kromasil C_4_ (Eka Chemical AB, Bohus, Sweden). The amount of loaded sample was 500 mg and the column volume was 400 mL (solvent A: 0.1% TFA; solvent B: 99% ACN and 0.1% TFA. Elution was carried out at a flow rate of 30 mL/min at 40°C with a linear gradient of ACN (30–80%) for 145 min. The volume of each fractionation was 75 ml. Absorbance was measured at 280 nm.

### Analytical methods

The cell density of the culture was calculated by reading the absorbance at 600 nm. The concentration of glucose in the culture broth was monitored using a glucose analyzer (YSI 2700 STAT, YSI, Yellow Springs, OH). The expression of rhG-CSF was analyzed by gradient sodium dodecyl sulfate-polyacrylamide gel electrophoresis (SDS-PAGE; 4–12% NuPAGE®, Bis-Tris Gel; Invitrogen, Grand Island, NY). The gels were visualized using Coomassie brilliant blue staining methods as previously shown [25]. The molecular weight markers were Precision Plus Protein All Blue Standard, SeeBlue Pre-Stained Standard from Bio-Rad (Hercules, CA), or Novex by Life Technologies (Grand Island, NY). Then, the sample was further analyzed by a RP C_4_ column (250 × 4.6 mm, Bio-Rad) with modified method [26]. Elution was carried out at a flow rate of 0.8 mL/min at 40°C with a linear gradient of ACN (42–76%) for 75 min. Absorbance was measured at 215 nm. Isoelectric focusing (IEF) was also performed using a pH 3–10 IEF gel (Invitrogen, Grand Island, NY). Protein concentration was measured by the Bradford method [27].

### Image analysis

After Coomassie brilliant blue staining, the gels were scanned using an ImageMaster VDS Gel Documentation System (Pharmacia Biotech, Uppsala, Sweden). Image analysis was performed using ImageMaster VDS analysis software (Pharmacia Biotech). Spot intensity was quantified automatically by calculation of spot volume following normalization of the image by taking the ratio of intensity of one spot to total spots in each lane on the gel, and expressed as a fractional intensity (%).

### Characterization of rhG-CSF

The N-terminal amino acid residues of rhG-CSF were confirmed by a protein sequencing system (PE Biosystems, Foster City, CA) using the Edman degradation procedure. As well, gel permeation chromatography (GPC) was performed using a TSK-GEL G3000SW column (300 mm × 7.8 mm; TOSOH, Tokyo, Japan). The peaks were shown using a photodiode array detector measuring absorbance at 280 nm. Matrix-assisted laser desorption/ionization-time of flight (MALDI-TOF) mass spectrometry (Bruker Daltonik, Bremen, Germany) was used to determine the molecular mass of rhG-CSF protein. A 10 μL aliquot of the protein solution in 0.1% TFA was mixed with 10 μL of a 10 mg/mL 3,5-dimethoxy-4-hydroxycinnamic acid solution in 70% ACN and 0.03% TFA. The resulting solution was spotted on the MALDI target plate. External calibration was performed with a ProteoMass™ Protein MALDI-MS calibration kit (Sigma, St Louis, MO). Mass spectra in the m/z range of 5000–50,000 were acquired in the positive ion mode.

Purified and standard G-CSF (Filgrastim; Amgen, Thousand Oaks, CA) were reacted respectively with endoproteinase Glu-C for peptide mapping; the proteins were digested at a ratio of 1:5 of Glu-C enzyme to protein substrate. The mixture was incubated at 25°C for 18 h, stopped with the addition of 3% phosphoric acid, and then analyzed by RP-HPLC [28].

### Western blotting

Both standard and purified rhG-CSF (5 μg) deployed on 4-12% SDS-PAGE were transferred to a nitrocellulose membrane, and the membrane was incubated for 1 h at room temperature (RT) with blocking buffer [50 mM sodium phosphate, 150 mM sodium chloride pH 7.0 buffer, and 0.05% Tween 20 (PBST)] containing 5% (w/v) dried skim milk. Subsequently, the membrane was incubated with an anti-hG-CSF polyclonal antibody (1:1000 dilution; Santa Cruz Biotechnology, Dallas, TX) overnight at 4°C in blocking buffer. The blot was then incubated with a secondary antibody conjugated with horseradish peroxidase (1:1000 dilution) for 2 h at RT. Diaminobenzidine (DAB) was used to develop the signal. The molecular weight marker was Precision Plus Protein Dual Color Standard (Bio-Rad, Hercules, CA).

### Biological activity of rhG-CSF

The bioactivity of rhG-CSF was analyzed by its ability to stimulate the proliferation of the murine myeloblastic NFS-60 cell line [29]. G-CSF-dependent NFS-60 cells dispersed in RPMI 1640 medium containing 200 mM l-glutamine and 1 mM Na-pyruvate at a concentration of 5.0 × 10^3^ cells/well in a 96-well plate (SPL, Pocheon, Korea). The standard G-CSF (NIBSC code: 09/136) was from the World Health Organization (WHO) 2^nd^ International Standard (NIBSC, Hertfordshire, UK). Both standard G-CSF and purified rhG-CSF were serially diluted 2-fold in growth medium and added to the well at a final concentration of 1,600 pg/mL and 3.12 pg/mL, respectively, in a total volume of 100 μL/well. The culture plate was incubated in a 37°C humidified CO_2_ incubator with 5% CO_2_ for 48 h. The cell proliferation reagent WST-1 (Roche Applied Science, Indianapolis, IN) was added and the cells were incubated for an additional 4 h. Absorbance was determined at 450 nm using an microplate reader (Bio-Tek, Winooski, VT). The absorbance was directly proportional to the number of viable cells because the tetrazolium salts in the WST-1 are cleaved to formazan by mitochondrial dehydrogenases in the cells. As well, the activity of rhG-CSF was measured using a Quantikine hG-CSF enzyme-linked immunosorbent assay (ELISA) kit (R&D Systems, Minneapolis, MN) according to the manufacturer’s protocol.

### Statistical analysis

The student t-test was used to assess significant differences between the treatment groups. The criterion for statistical significance was set at *p*<0.05.

## Results

### Expression of rhG-CSF in *E. coli*

**Figure 1:**
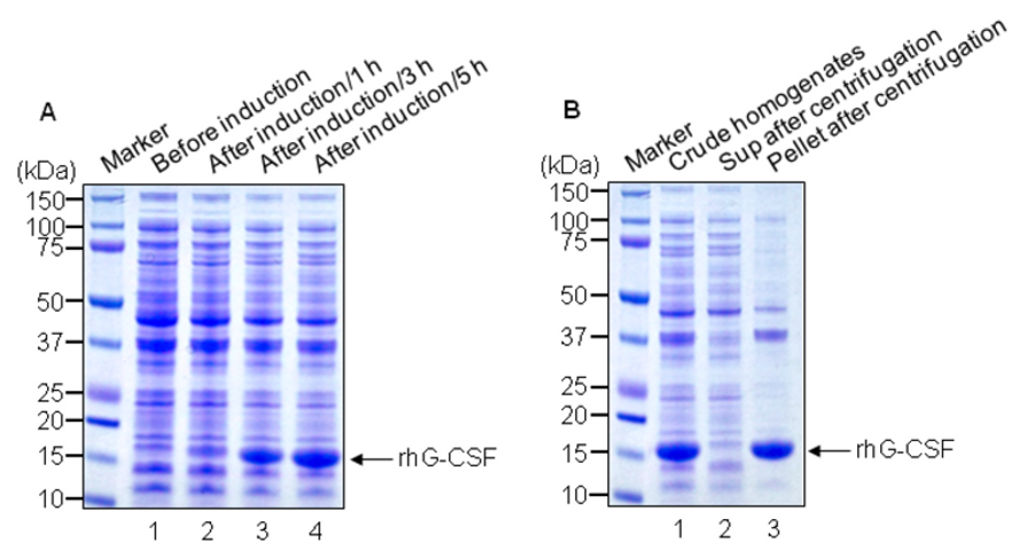
Analysis of rhG-CSF protein by SDS-PAGE. Upon IPTG induction, rhG-CSF was analyzed using a 4–12% reducing SDS-PAGE gel followed by Coomassie brilliant blue staining. **A.** Lane 1, cell homogenates of *E. coli* JM109/pPT-G-CSF without IPTG induction; lane 2, cell homogenates after IPTG induction for 1 h; lane 3, After IPTG induction for 3 h; lane 4, After IPTG induction for 5 h. **B.** Lane 1, total homogenates; lane 2, supernatant after centrifugation; lane 3, Pellet after centrifugation. Most of IPTG induced rhG-CSF is pelleted after centrifugation. The arrow indicates rhG-CSF.

To express rhG-CSF molecules in *E. coli* JM109, the human *g-csf* gene was cloned into a pPT plasmid. Batch culture with *E. coli* JM109/pPT-G-CSF was carried out in a flask containing 50 mL of the initial medium at 250 rpm, 37°C. As shown in Figure 1A, rhG-CSF expression was started at 3 h after IPTG induction, lasted for 5 h (lane 3-4). However, most of the rhG-CSF was observed in pellets after centrifugation (Figure 1B, lane 3). The molecular weight of rhG-CSF was shown at approximately 18-kDa location. Densitometric analysis by imaging system indicated that rhG-CSF out of the total proteins comprises the content of 41.3%.

### Fed-batch culture

**Figure 2:**
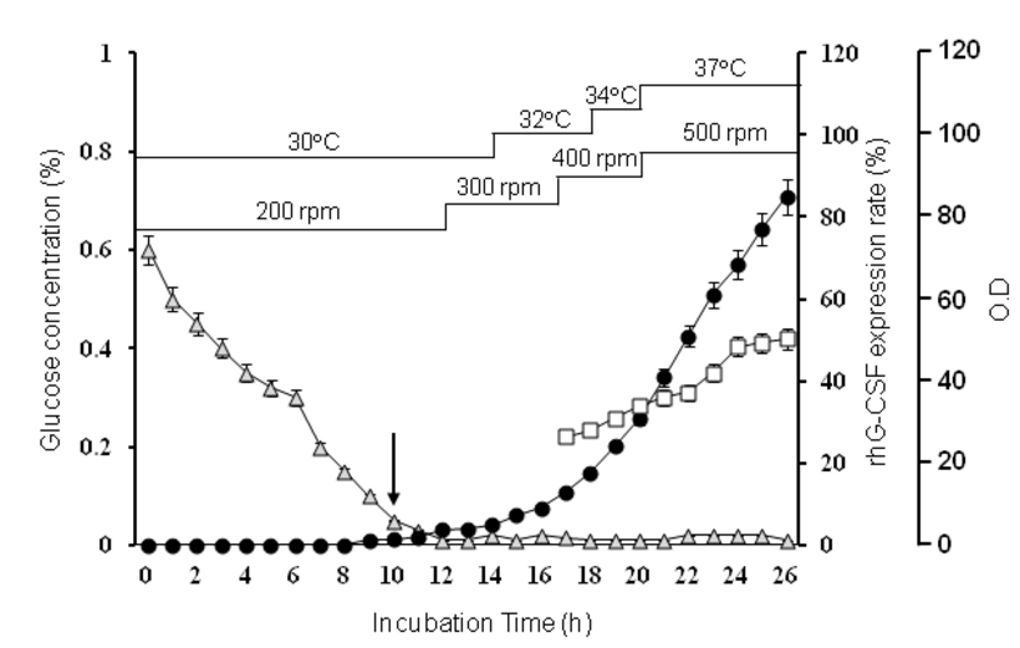
Cell growth and rhG-CSF expression during the fed-batch culture. *E. coli* JM109/pPT-G-CSF cells were grown in fed-batch culture using temperature shift method in a 300-L fermentor with glucose as the energy source. Optical density was detected using a spectrophotometer at 600 nm. Glucose concentration (grey triangle); OD_600_ (●); Expression rate of rhG-CSF (□). The arrow indicates the start time of feeding.

Because *E. coli* JM109 were transformed properly by pPT-G-CSF plasmid, the stepwise increase strategy of temperature for the protein expression was performed with the same cells [23,24]. As shown in Figure 2, the growth pattern of the cells and rhG-CSF production in fed-batch culture were increased by time-dependent manner. It was first carried out at 30°C, and then increased in a stepwise to 37°C. The addition of feeding solutions A and B led to the exponential growth of the cells, showing optical density of 84.92 at 26 h. The expression of rhG-CSF was first detected at 17 h, lasted along with cell growth. Through this culture, 42.8 g of dry cells per liter of culture broth were obtained. The expression rate of rhG-CSF out of the total proteins was 50.4%.

### Refolding of hG-CSF

**Figure 3:**
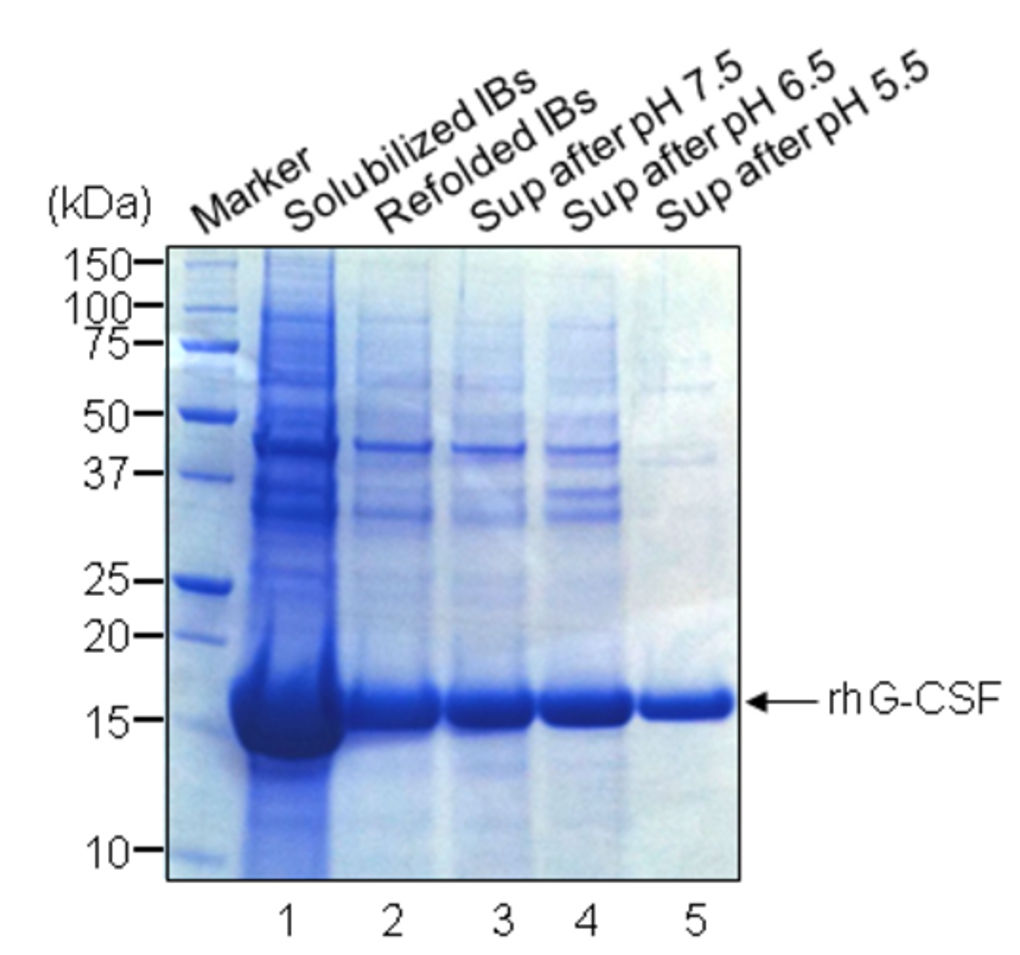
The purity of rhG-CSF is increased following refolding from inclusion bodies. Insoluble fraction of the induced culture were harvested, refolded, and precipitated under the stepwise decrease of pH (7.5→5.5). The refolded samples before and after pH precipitation process were analyzed by reducing 4-12% SDS-PAGE, followed by Coomassie brilliant blue staining. Lane 1, solubilized IBs; lane 2, refolded IBs before pH precipitation; lane 3, supernatant (Sup) after pH 7.5 precipitation; lane 4, sup after pH 6.5 precipitation; Lane 5, sup after pH 5.5 precipitation. The arrow indicates rhG-CSF.

The most of rhG-CSFs were detected in IBs of cultured *E. coli* JM109. To refold, the IBs in the culture broth were harvested by centrifugation after the cells were disrupted. The harvested IBs were washed with a buffer (2 M urea, 0.1 M Tris-HCl, 0.5% Triton X-100, and 0.02% lysozyme pH 7.0) and refolded in a dilution step to decrease the concentration of urea. To precipitate impurities containing misfolded rhG-CSF, lipids, and other unnecessary proteins, the pH of the refolded rhG-CSF solution was stepwise lowered from 9.5 to 3 by the addition of phosphoric acid [23]. The optimum precipitation of the refolded solution, in terms of purity and yield, was pH 5.5. Figure 3 shows the SDS-PAGE analysis of refolded rhG-CSF before and after pH precipitation between 7.5 and 5.5, resulting in the removal of a majority of impurities; at pH 5.5 (lane 5). The recovery rate of refolded rhG-CSF was approximately 70%. However, large quantities of rhG-CSF were still co-precipitated. In spite of these **losses**, pH precipitation process was positively necessary because the purity of rhG-CSF was increased from 70% to 85% based on image analyses. Subsequently, both unrefolded and refolded rhG-CSF were analyzed by RP-HPLC. As a result, the retention time of rhG-CSF before and after refolding was shifted from 54.3 min to 52.5 min (data not shown), suggesting that the refolded form is more hydrophilic. The refolding yield was increased to 65% by RP-HPLC.

### Prep-HPLC

**Figure 4:**
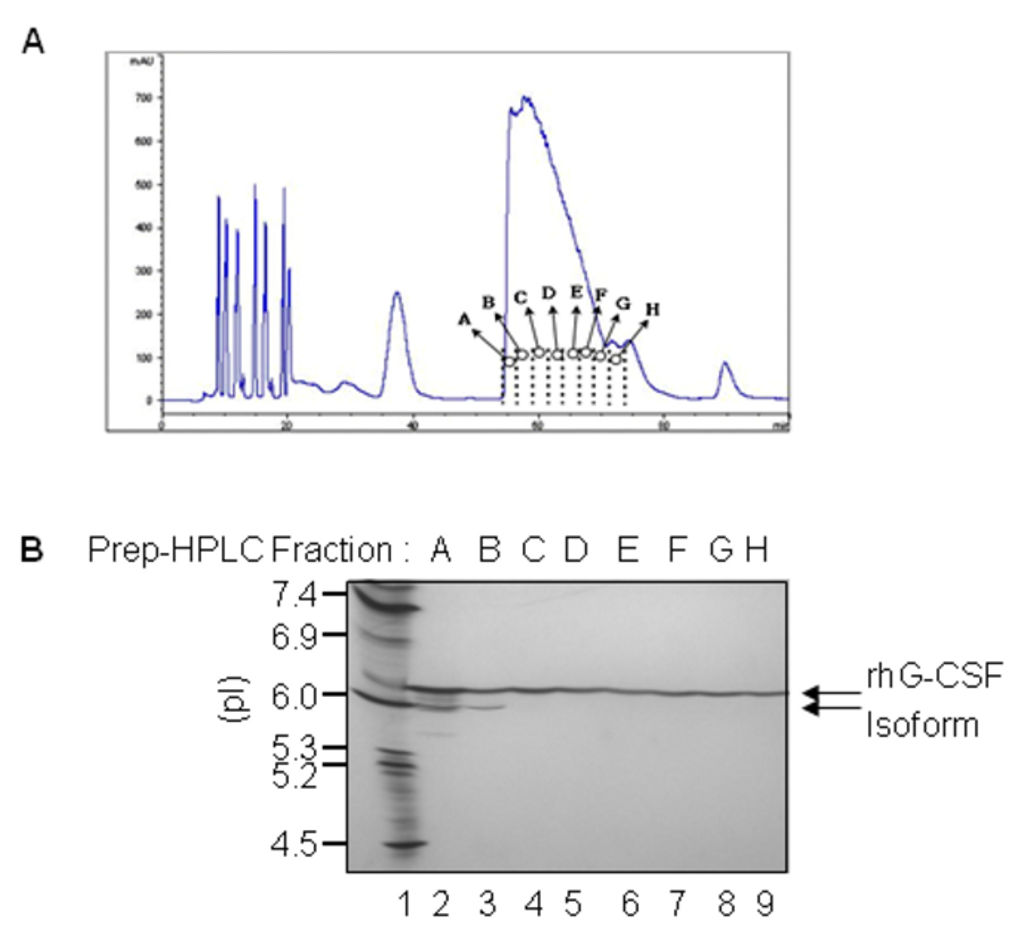
Prep-HPLC chromatogram and IEF analysis. Prep-HPLC chromatography was performed to detect rhG-CSF protein. **A**. Chromatogram of prep-HPLC with the sup after pH precipitation of refolded rhG-CSF. **B**. IEF analysis of each fraction from A to H obtained by Prep-HPLC. The IEF marker indicates pI. Lane 1, pI marker 4.5–7.4. Absorbance is in milliabsorbance units (mAU).

It is not easy to separate the isoforms of rhG-CSF from homogenates because their physicochemical properties are quite similar each other. Although there was an attempt to separate isoforms from rhG-CSFs by two cationic exchange chromatography steps [18], it is actually uneconomical because of tedious processes and low yields. Consequently, we tried with a modified method using pH precipitation method. The supernatant obtained through pH precipitation of the refolded sample was concentrated by an ultrafiltration system, loaded onto a prep-HPLC column, and sample fractions (A to H) were collected from the column at 2.5-minute intervals (Figure 4A). Each fraction was analyzed on IEF gel (Figure 4B). Interestingly, the isoforms of rhG-CSF were eluted in front of the main peak (lane 2 and 3). Therefore, fractions C to H were pooled except fractions A and B, concentrated, and analyzed by RP-HPLC. Our result suggests that only a prep-HPLC step after pH precipitation may be sufficient to separate the isoforms. The average yield and purity in each purification step are summarized in Table 1. It is shown that the each yield of the acidic precipitation, ultrafiltration, and prep-HPLC was 69%, 86%, and 76%, respectively, which was quite similar to those that were obtained from small-scale purification [22,30]. 1.75 g of rhG-CSF was obtained from 1 L culture broth.

**Table 1:** Summary of each step during the purification of rhG-CSF.

Process Steps	Total Mass (gram/Mean±S.D.*)	Yield	Purity (%)	Remark Weight
Cell Paste	134±6.7	-	-	Amount of Water content: 80%
Washed IB	47.5±1.9	-	-	Amount of Water content: 65%
Refolding	3.87±0.19	65	70	RP-HPLC & SDS-PAGE
Acidic Precipitation	2.69±0.094	69	85	RP-HPLC & SDS-PAGE
Ultrafiltration	2.31±0.035	86	87.5	RP-HPLC
Prep-HPLC	1.75±0.079	76	99	RP-HPLC

* Each value represents the mean of five performances ± S.D.

### Characterization of rhG-CSF

**Figure 5:**
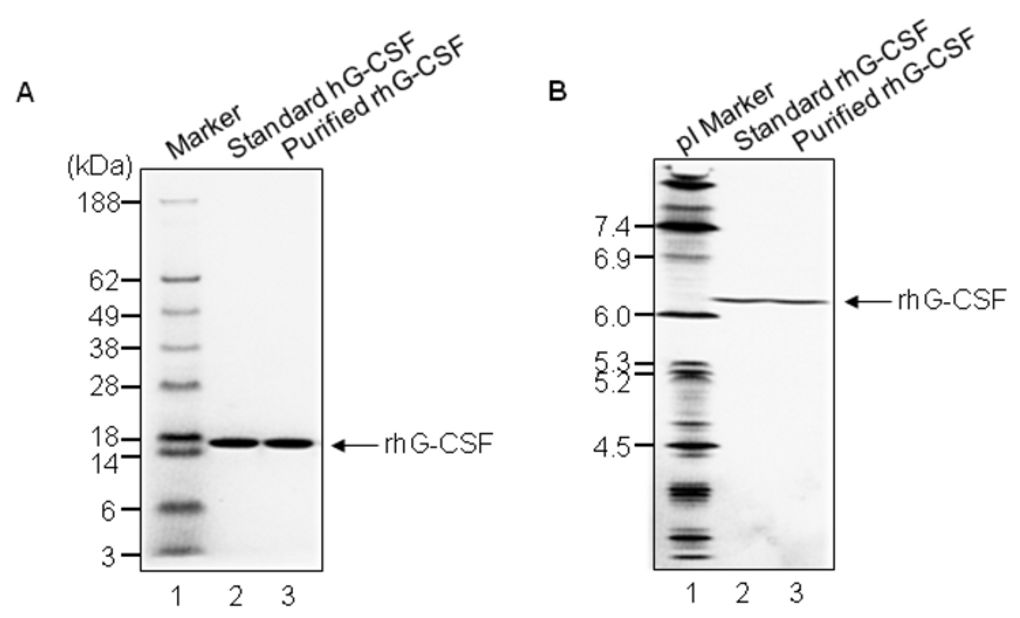
The analysis of rhG-CSF protein by SDS-PAGE and IEF. **A**. A 4–12% discontinuous NuPAGE SDS-PAGE gel and Coomassie brilliant blue staining were used to confirm the purity of rhG-CSF. Lane 1, molecular weight marker; lane 2, standard rhG-CSF (Filgrastim); lane 3, rhG-CSF. **B**. The Novex pH 3–10 IEF gel was used to examine the purity of rhG-CSF. The IEF marker indicates pI. Lane 1, pI marker 4.5–7.4; lane 2, standard hG-CSF; lane 3, purified rhG-CSF. The arrow indicates rhG-CSF.

**Figure 6:**
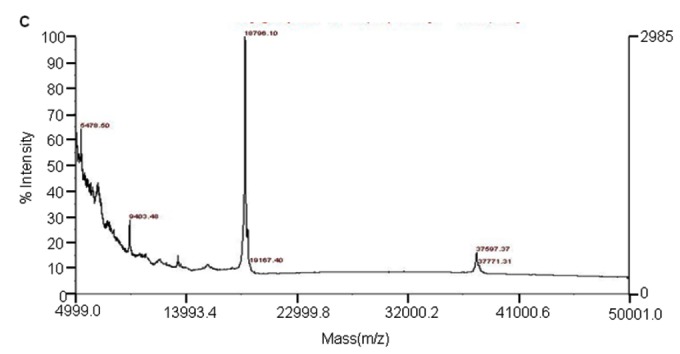
rhG-CSF analyses. **A**. RP-HPLC chromatogram of rhG-CSF. Samples (30 μg) containing rhG-CSF for analysis was loaded on the RP-HPLC column. **B**. GPC-HPLC chromatogram of rhG-CSF. **C**. MALDI mass spectra of rhG-CSF.

The first 35 amino-terminal amino acid residues of rhG-CSF protein were in order of MTPLGPASSLPQSFLLKCLE-QVRKIQGDGAALQEK by the Edman degradation analysis, being in tune with the expected sequences. For purity analysis of rhG-CSF, SDS-PAGE analysis was performed, followed by Coomassie brilliant blue staining. As shown in Figure 5A, rhG-CSF (lane 3) stands line abreast with standard hG-CSF (lane 2, Filgrastim) between 18 kDa and 14 kDa. Next, two proteins were analyzed by a pH 3–10 IEF gel. As shown in Figure 5B, the two proteins were observed at the same location on the gel (lanes 2 and 3). The pI of two proteins was near 6.1, implying that they are the same rhG-CSFs. No other isoform of rhG-CSF was detected, meaning that the rhG-CSF exists in a condition of high-purity. An RP-HPLC chromatogram of rhG-CSF showed that it was eluted as a single peak at approximately 30 min without any contamination (Figure 6A). The purity of rhG-CSF was measured to be approximately 99%. To confirm the molecular weight of the rhG-CSF, GPC-HPLC was performed additionally (Figure 6B). Through the GPC-HPLC, it was estimated at approximately 19.0 kDa (expected value: 18.733 kDa) since a peak was found with a retention time of 17.8 min. Only one major peak for rhG-CSF was detected. The molecular mass of rhG-CSF was determined to be 18.796 kDa by the analysis of MALDI-TOF mass spectrometry; this value was close to the expected mass of 18.733 kDa (Figure 6C).

**Figure 7:**
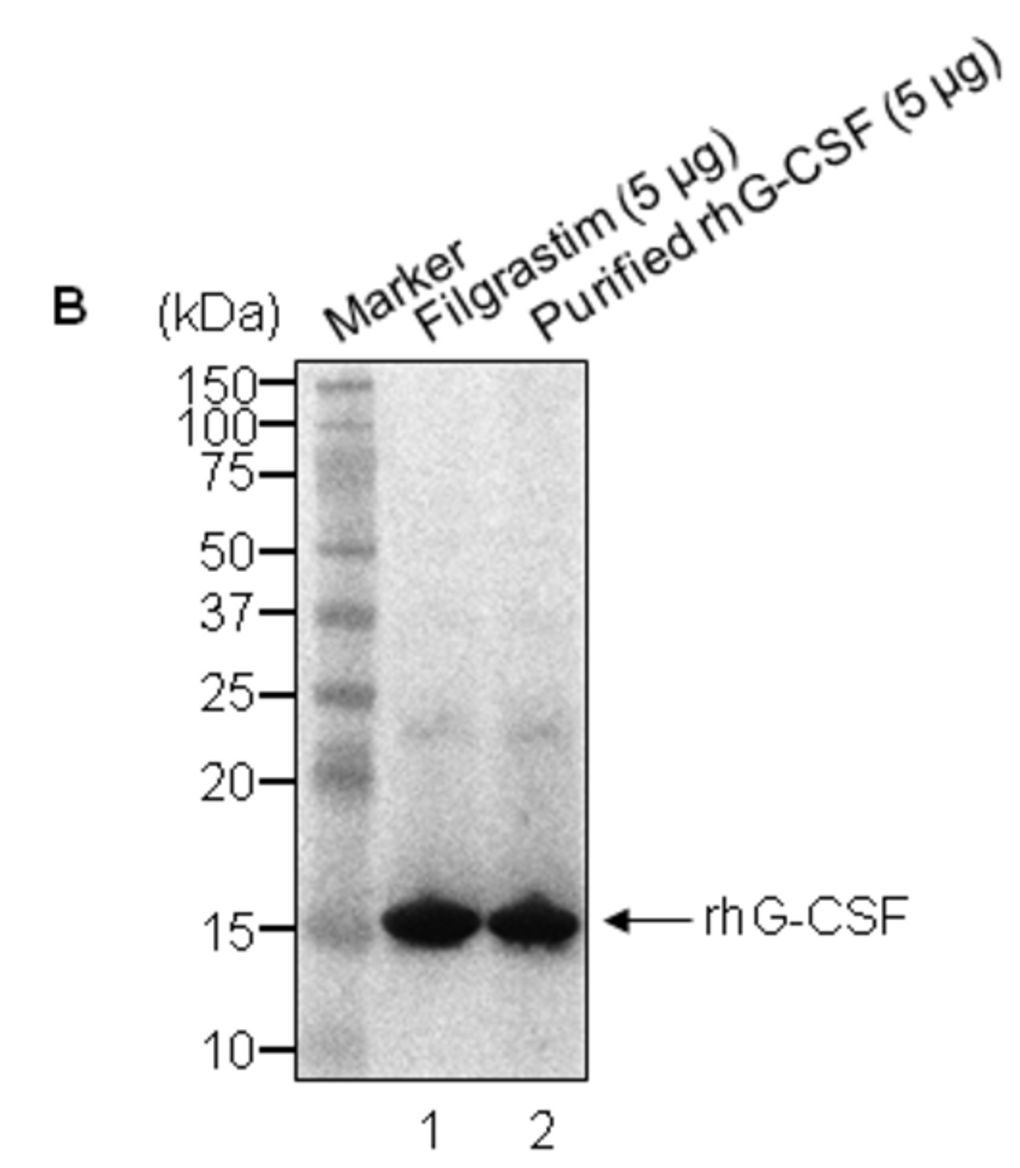
Peptide map and Western blot analysis of standard hG-CSF and purified rhG-CSF. **A**. Two chromatograms of standard hG-CSF and rhG-CSF are overlapped for comparison. The solid arrow is the chromatogram for rhG-CSF and the dotted arrow is the chromatogram for standard hG-CSF. Absorbance is in absorbance units (AU). **B**. Two rhG-CSF proteins were examined by western blot after a 4–12% reducing SDS-PAGE performance. Lane 1, standard rhG-CSF (Filgrastim, 5 μg); lane 2, purified rhG-CSF (5 μg). The arrow indicates rhG-CSF.

The rhG-CSF was digested with endoproteinase Glu-C and analyzed by a C18 RP-HPLC column. The peaks of rhG-CSF and standard hG-CSF were overlapped in Figure 7A. Their chromatograms were shown quite similar patterns which are undistinguishable to the naked eye. As well, in western blot analysis, a single band of rhG-CSF was detected by anti-hG-CSF polyclonal antibodies as the filgrastim was done (Figure 7B, lanes 1 and 2).

### Biological assay

**Figure 8:**
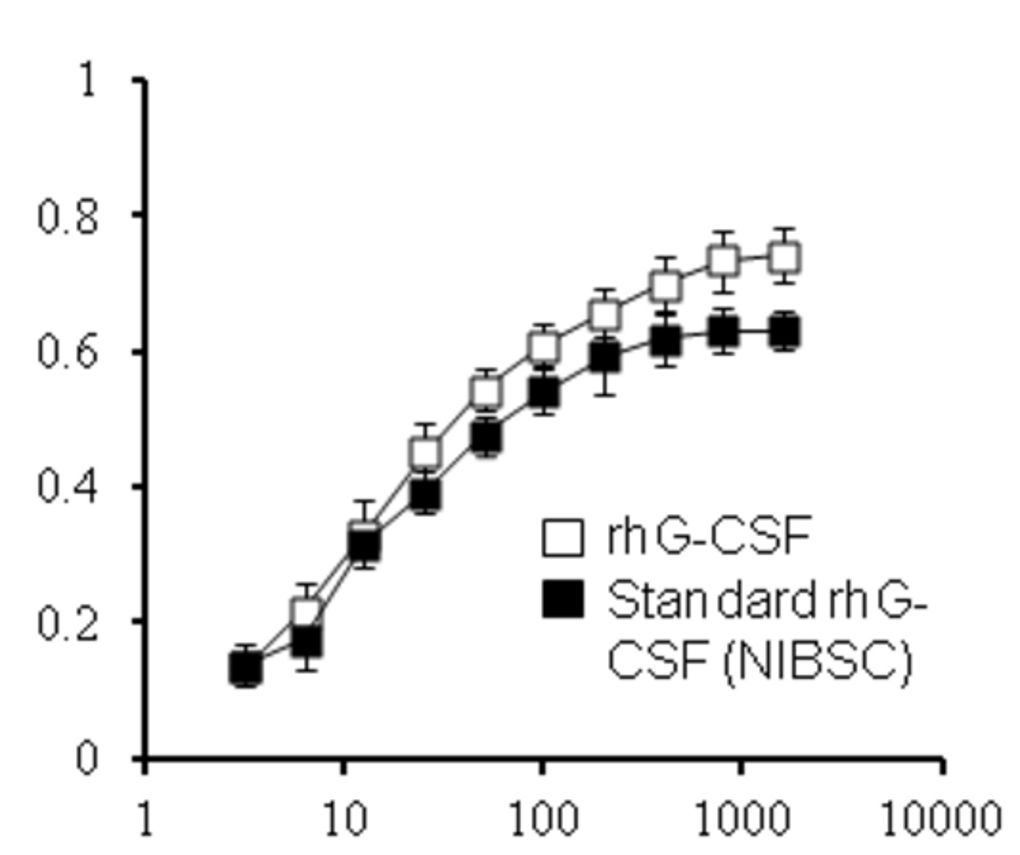
Biological activity of rhG-CSF protein. The rhG-CSF protein was assessed for its ability to stimulate the proliferation of an NSF-60 cells. The cells were incubated for 48 h in the presence of rhG-CSF. As a control, standard hG-CSF (WHO 2^nd^ International Standard) was also analyzed. The bioactivity of rhG-CSF on NFS-60 cell proliferation was measured using the reagent WST-1. Data are the mean ± SD of triplicate measurements (significant versus control, p<0.05).

To test the biological activity of rhG-CSF, we carried out a proliferation assay with NFS-60 cells. The cells were treated with various concentrations of standard or rhG-CSF for 48 h. Their activity was then assessed using a reagent WST-1. As shown in Figure 8, rhG-CSF promoted 50% maximal growth of the cells at a concentration of 13.4 pg/mL. Standard hG-CSF had also comparable activity at 13.9 pg/mL, drawing a conclusion that both have similar biological activities. The activity of rhG-CSF was calculated to be 1.0 × 10^8^ IU/mg according to ELISA and the cell proliferation assay.

## Discussion

In this study, we established the new method for the industrial-scale production and characterization of rhG-CSF. pET expression system has been extensively used for the production of rhG-CSF in *E. coli* [26,31,32]. Meanwhile, because we have already accumulated technology for the efficient expression system of a target gene, using fed-batch culture [23,24], we chose pPT expression system containing the growth rate-dependent *rrnB* P2 promoter instead of pET system, [13,23,33]. A method using stepwise temperature shifts (30°C→37°C) was also selected on the basis of previous study [23]. This method without inducers such as IPTG is established on a buffered medium that contains glucose as carbon sources. In this study, the expression of rhG-CSF by temperature shifts allowed over 10-fold higher cell densities when compared to that under IPTG induction [22,32]. However, this was slightly lower than that of Khalilzadeh et al. obtained by 64g/L [34]. This discrepancy might be caused by difference of total volumes (120-L vs 2-L).

High-level expression of rhG-CSF in *E. coil* leads to the unavoidable result of IBs, which should be solubilized in chaotropic agents such as high grade GuHCl or urea [15,26,34]. Recently, it was reported that the usage of high-grade GuHCl is necessary to achieve high solubility and efficient refolding [15]; however, GuHCl is uneconomical because of high-costs. It was here demonstrated that urea may be efficient for solubilizing and refolding of IBs, as washing of IBs with urea improves the refolding yield and purity of rhG-CSF. This is inconsistent with Thomson et al who reported that extensive washing of IBs is not necessary to improve the refolding yield [15].

The function of ultrafiltration is to remove contaminants as well as to reduce volume before the refolded sample passes to an ion exchanger chromatography. Linear scale flat-sheet cassettes were chosen in this pilot-scale purification. The main advantages of this module are that they are suitable for bioprocessing industry that provide 1000-fold scaling up of ultrafiltration processes with consistent product yield and process flux [35]. However, the disadvantage of the flat-plate design is that they are susceptible to particulate plugging and the modules are difficult to clean. In addition, there is another problem; it is a price.

There are several reports related to the purification of rhG-CSF [20,22,36,37]. Among them, Gomes et al reported that a single step is sufficient to obtain a highly purified mutant form of rhG-CSF in *E. coil* [37]. Although their research gives a lot of useful informations regarding rhG-CSF production, we are specially interested in knowing if isoforms (by IEF gel) during the purification were detected. Our results obviously demonstrate that the isoform of rhG-CSF can be separated in the early fractions of the prep-HPLC step (Figure 4B).

Figure 4A indicates that the isoform of rhG-CSF have higher hydrophilicity. This is consistent with Hinderer et al reported previously [19]. Also, Lu et al reported that three kinds of isomers have high bioactivity *in vitro* and slightly lower pI`s [18]. Although the isoforms in this study were not further characterized, it is presumed to be in three kinds of isoforms. Through this study, the yield obtained was 1.75g/L of rhG-CSF, and the purity was 99%. In spite of overall steps by large scale, our results show that the yield and purity of rhG-CSF are of the highest grade when compared with the other reports performed at small-scale [15,22,26].

Regarding the biological activity of purified rhG-CSF, it showed that rhG-CSF promotes 50% maximal growth of the cells at 13.4 pg/mL. It proves that they have extremely similar activities when compared with that of World Health Organization (WHO) 2^nd^ International Standard. Above all, it is emphasized that new method for purification of rhG-CSF was cost-effective one because the overall costs of expression were reduced by omitting the addition of IPTG.

## Conclusion

Here, we describe a detailed report about simplified large-scale refolding, purification, and characterization of rhG-CSF in *E. coli*. First, in the fed-batch culture, recombinant protein expression by a temperature shift strategy without inducer such as IPTG is a compatible method for its large-scale production. Second, the purification by a single step of prep-HPLC after the pH precipitation of the refolded samples is sufficient for the industrial-scale production. In addition, our results show that the isoforms of rhG-CSF can be separated during the prep-HPLC step. Taken together, results described here demonstrate that experimental methods and strategies described here to generate high-purity recombinant proteins may be worthful for the large-scale production.
